# A Retrospective Longitudinal Study of Refugees With Eosinophilia at an Academic Center in the United States From 2015 to 2020

**DOI:** 10.1093/ofid/ofae430

**Published:** 2024-07-19

**Authors:** Ann Ding, Marcela Osorio, Maranatha Teferi, Benjamin Gallo Marin, Margarita Cruz-Sánchez, Matthew Lorenz, Ian C Michelow

**Affiliations:** Warren Alpert Medical School of Brown University, Providence, Rhode Island, USA; Department of Pediatrics, Rhode Island Hospital, Providence, Rhode Island, USA; Warren Alpert Medical School of Brown University, Providence, Rhode Island, USA; Warren Alpert Medical School of Brown University, Providence, Rhode Island, USA; Warren Alpert Medical School of Brown University, Providence, Rhode Island, USA; Warren Alpert Medical School of Brown University, Providence, Rhode Island, USA; Warren Alpert Medical School of Brown University, Providence, Rhode Island, USA; Department of Pediatrics, Rhode Island Hospital, Providence, Rhode Island, USA; Department of Pediatrics, University of Connecticut School of Medicine, Farmington, Connecticut, USA; Division of Infectious Diseases and Immunology, Connecticut Children's, Hartford, Connecticut, USA

**Keywords:** eosinophilia, health screening, parasites, refugee, United States of America

## Abstract

**Background:**

Refugees to the United States frequently have parasitic infections. If untreated, parasites can cause severe complications. The purpose of this study was to investigate the incidence, management, and outcomes of eosinophilia as a biomarker for parasites.

**Methods:**

We conducted a retrospective longitudinal chart review of consecutive refugees attending 3 refugee clinics in Rhode Island that manage the health care of all pediatric and adult refugees.

**Results:**

Among 812 refugees who met inclusion criteria, 147 (18.1%) had eosinophilia upon arrival and almost half had ≥1 symptom. The rates and severity of eosinophilia in those with predeparture presumptive treatment records who did (112/115, 97.4%) or did not (488/498, 98.0%) receive predeparture antiparasitic treatment were similar. All refugees with eosinophilia had ≥1 parasitic test in the United States. The most common attributable parasites were *Schistosoma* and *Strongyloides stercoralis*. Overall, parasites were detected in 63 (42.9%) of 147 refugees with eosinophilia by either stool testing, serology, or blood smear, but testing was inconsistent and likely underestimated true incidence. Only some of the identified parasites typically cause eosinophilia. Forty-five (30.6%) refugees with eosinophilia received antiparasitics in the United States. Of 81 (55.1%) individuals who had repeat blood tests, eosinophilia had resolved in 52 (64.2%). Five individuals (3.4%) had alternative diagnoses, including eczema, myelofibrosis, and drug allergy.

**Conclusions:**

Our findings support Centers for Disease Control and Prevention recommendations to screen for eosinophilia in newly arrived refugees. Follow-up after 3–6 months is critical to confirm resolution of residual eosinophilia, which frequently occurs after effective predeparture treatment or if eosinophilia persists, to diagnose active parasitic infections.

High rates of parasitic pathogens have been identified in asymptomatic, newly arrived refugees to the United States [1–3]. Left untreated, parasitic infections can have significant health consequences including anemia, malnutrition, neurocognitive deficits, infertility, urinary tract malignancy, and death [1, 3, 4]. Many helminths such as *Ancylostoma duodenale* and *Necator americanus* (hookworms), filariae, *Strongyloides stercoralis*, *Schistosoma* spp., and *Toxocara* spp., among others, may cause eosinophilia (absolute eosinophil count >450/μL). Thus, eosinophilia has been identified as a potential biomarker for parasitic infections, among other health conditions. However, its predictive value for parasitic infections varies widely because it is not specific to parasites and many pathogenic parasites do not cause eosinophilia [2, 4–7].

According to Centers for Disease Control and Prevention (CDC) guidelines, refugees should receive presumptive therapy for parasitic infections before their arrival in the United States (Supplementary Table 1) [8]. Refugees, however, may receive incomplete antiparasitic regimens depending on drug availability and patient adherence, or no therapy if contraindicated or if the country of exit has yet to implement a treatment program. Consequently, some parasites may not be effectively treated. Among the common causes of eosinophilia, albendazole targets soil-transmitted helminths such as *Ascaris lumbricoides*, *Trichuris trichiura*, and *Ancylostoma duodenale* and *Necator americanus* (hookworms), whereas ivermectin primarily treats *Strongyloides stercoralis* and praziquantel targets *Schistosoma* species.

CDC guidelines recommend that refugees undergo a domestic medical examination (DME) 30–90 days after arrival to the United States. Not infrequently, individuals who received effective predeparture presumptive antiparasitic treatment may still have lingering eosinophilia, also known as residual eosinophilia. In that scenario, absolute eosinophil counts should be re-evaluated 3–6 months later to assess resolution. If eosinophilia persists thereafter, a specific diagnosis should be pursued, as described in Figure 1 of the CDC Intestinal Parasites website [9].

**Figure 1.**
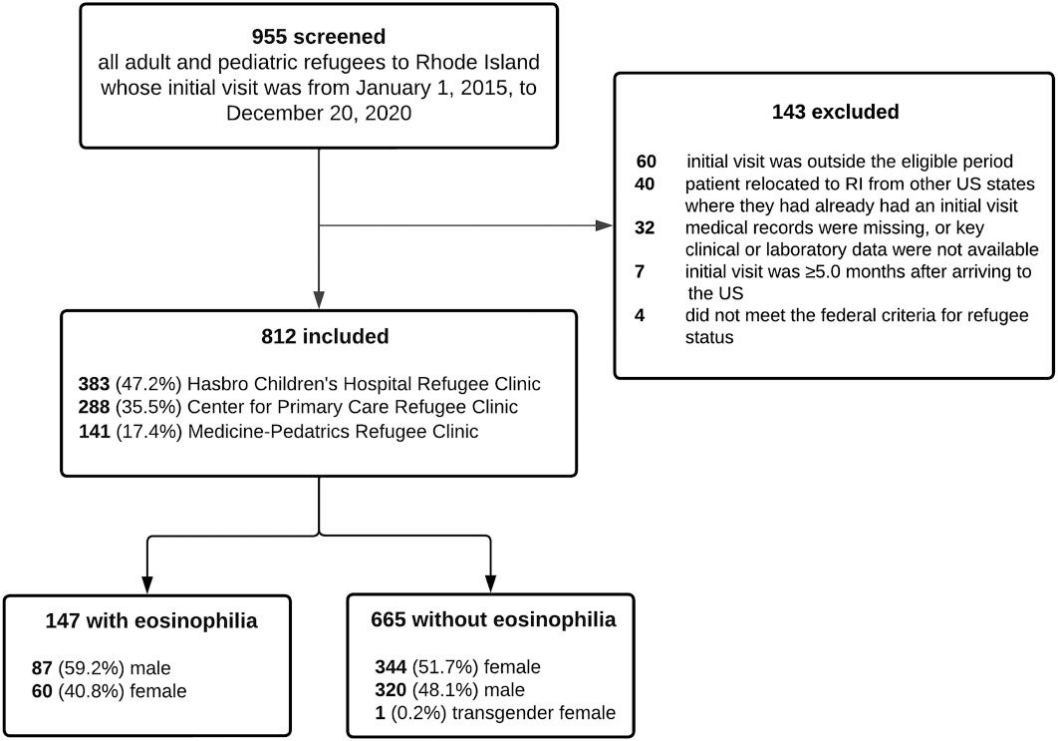
Study flow diagram.

Eosinophilia observed postarrival may be caused by a host of infectious and noninfectious disorders [10], and practice patterns for managing eosinophilia in refugees arriving to the United States vary widely. Management may include simply monitoring eosinophilia counts without targeted investigation or therapy for 3–6 months, screening for symptoms of parasitic infections and selectively offering diagnostic tests and treatment, routinely adopting a “test and treat” approach with 2 or more separate stool ova and parasite tests, serologic testing for certain parasites (eg, *Schistosoma, Strongyloides*), or administering presumptive antiparasitic agents regardless of symptoms [9]. Clinicians who perform the DME may differ from those providing ongoing medical care for new arrivals, which may affect clinical decision-making regarding eosinophilia (eg, repeating empiric antiparasitic treatment or monitoring for resolution). Considering that time to resolution of eosinophilia after effective treatment may vary and success of antiparasitic treatment is not 100%, interpreting persistent eosinophilia may be challenging [8, 11]. Furthermore, many other medical conditions may be associated with eosinophilia including allergies (environmental, food, or drug), asthma, atopic dermatitis, immunodeficiency, nonparasitic infections, sickle cell disease, and rarely hematological malignancies [10].

Resettled refugees, a vulnerable population that faces significant barriers in accessing health care, may struggle with following up for monitoring eosinophilia. Additional challenges include the numerous potential etiologies of eosinophilia and US providers’ general lack of experience with diagnosing and managing certain parasitic diseases such as strongyloidiasis or schistosomiasis [9]. Current CDC guidelines provide general region-specific recommendations for screening and treating the most common parasitic infections in refugees. If eosinophilia persists and a parasitic cause has not been ruled out, health care providers are advised to consult Infectious Diseases experts [8, 9].

Previous studies have reported the incidence of eosinophilia and parasitic infections in refugees both before and after arrival in the United States [1, 2, 7, 12]. However, to our knowledge, there have been no recent US-based systematic investigations of refugees with eosinophilia, and no studies have analyzed their long-term clinical outcomes. The purpose of this study was to investigate the incidence, management, and outcomes of eosinophilia in newly arrived refugees presenting to specialized refugee clinics at a single academic medical center in Rhode Island, taking into account whether they received predeparture presumptive treatment.

## METHODS

### Study Design

A retrospective longitudinal chart review was performed at Rhode Island Hospital (RIH), Hasbro Children's Hospital, and The Miriam Hospital (Lifespan Inc. academic complex) in Providence, Rhode Island. The refugee clinics at these centers manage the health care needs of all pediatric and adult refugees who are resettled in Rhode Island.

### Inclusion and Exclusion Criteria

Consecutive pediatric and adult refugees who had their initial intake visit at the Center for Primary Care Refugee Clinic, Hasbro Children Hospital's Refugee Clinic, or the Medicine-Pediatric Refugee Clinic from January 1, 2015, to December 20, 2020, were eligible for inclusion.

Patients were excluded if (1) their initial visit was outside the eligible period, (2) medical records with key clinical or laboratory data were missing, (3) they relocated to Rhode Island from other US states where they initially presented, (4) the initial visit was ≥5.0 months after arriving in the United States, or (5) they did not meet the federal criteria for refugee status [13].

### Data Collection

Each clinic provided lists of refugees by searching electronic medical records, printed clinic records, or handwritten charts. Four investigators performed the retrospective chart review. Each reviewer initially extracted data from the charts of the same 5% of individuals with eosinophilia to assess inter-rater reliability, which was ≥90%. Demographic data and eosinophil counts were collected for all refugees. Further detailed epidemiological and longitudinal clinical and laboratory data were systematically collated for all subjects with eosinophilia. Data were entered and validated in a data collection survey using Research Electronic Data Capture (REDCap), a secure web-based application [14]. Discrepancies between reviewers or clinical inconsistencies discovered during the chart reviews were resolved by consensus.

In addition, 2 investigators collected predeparture information for all eligible refugees through the Centers for Disease Control and Prevention (CDC) Predeparture Electronic Disease Notification System to determine whether individuals were treated presumptively with antimicrobials or had received therapy for documented parasitic infections before departure from their country of exit [15].

### Laboratory Tests

All patients underwent initial laboratory testing in keeping with CDC recommendations for the DME, including complete blood cell and differential counts, performed at RIH hematology and clinical microbiology laboratories. Parasite blood smears and stool multiplex polymerase chain reaction (PCR) assays, also performed at RIH hematology and clinical microbiology laboratories, were ordered at the discretion of the provider. Stool ova and parasite tests and serum immunoglobulin assays for *Strongyloides stercoralis* and *Schistosoma* spp. were performed at the discretion of the provider at Associated Regional and University Pathologists (ARUP) Laboratories (Salt Lake City, UT, USA).

### Statistical Analysis

Data were analyzed using Microsoft Excel, version 16.72, and Statistical Package for Social Sciences (IBM SPSS Statistics, version 29.0; IBM Corp., Armonk, NY, USA). Descriptive statistics were used to analyze baseline characteristics using frequency and percentage for categorical data. The Pearson chi-square or Fisher exact test was used to assess associations between categorical variables, where appropriate. Two-tailed tests with *P* < .05 were considered statistically significant.

### Ethics Statement

The Lifespan Institutional Review Board approved the study and waived consent.

## RESULTS

### Selection of Study Subjects

We identified 955 eligible individuals for chart review. One hundred forty-three refugees were excluded for failing to meet inclusion criteria, resulting in enrollment of 812 subjects (Figure 1). Overall, 383 (47.2%) individuals were seen at the Hasbro Children's Hospital Refugee Clinic, 288 (35.5%) at the Center for Primary Care Refugee Clinic, and 141 (17.4%) at the Medicine-Pediatrics Refugee Clinic.

### Refugee Demographics

Almost half (48.4%) of the 812 enrolled refugees were children (age ≤18 years), and sexes were similarly represented (Table 1). Regions of origin and exit were defined by the World Health Organization classification [16]. The majority of refugees (62.2%) originated from Africa, and the next most frequent region was Asia (29.8%) (Table 1).

**Table 1.** Refugee Demographics Stratified by Eosinophilia

Characteristic	Eosinophilia, No. (%)	No Eosinophilia, No. (%)	All Refugees, No. (%)
Overall	147 (18.1)	665 (81.9)	812 (100.0)
Sex	…	…	…
Female	60 (40.8)	344 (51.7)	404 (49.8)
Male	87 (59.2)	320 (48.1)	407 (50.1)
Transgender female	0 (0.0)	1 (0.2)	1 (0.2)
Age	…	…	…
Children (≤18 y)	68 (46.3)	325 (48.9)	393 (48.4)
Adult (>18 y)	79 (53.7)	340 (51.1)	419 (51.6)
Continent of origin^a^	…	…	…
Africa	125 (85.0)	375 (56.4)	500 (61.6)
Asia	19 (12.9)	237 (35.6)	256 (31.5)
Europe	2 (1.4)	20 (3.0)	22 (2.7)
Americas	1 (0.7)	33 (5.0)	34 (4.2)
Australia	0 (0.0)	0 (0.0)	0 (0.0)
Continent of exit^b^	…	…	…
Africa	121 (82.3)	385 (57.9)	506 (62.3)
Asia	14 (9.5)	164 (24.7)	178 (21.9)
Europe	11 (7.5)	84 (12.6)	95 (11.7)
Americas	1 (0.7)	31 (4.7)	42 (5.2)
Australia	0 (0.0)	1 (0.2)	1 (0.1)

^a^Specific countries of origin: *Africa* (500): Burundi (23), Congo (37), Democratic Republic of Congo (151), Djibouti (1), Egypt (1), Eritrea (20), Ethiopia (6), Ivory Coast (3), Kenya (16), Liberia (12), Malawi (4), Mozambique (7), Namibia (2), Rwanda (14), Somalia (88), South Africa (6), Tanzania (82), Uganda (15), Zambia (10), Zimbabwe (2). *Asia* (256): Afghanistan (21), Bhutan (1), Iran (1), Iraq (59), Jordan (4), Malaysia (2), Nepal (6), Pakistan (1), Saudi Arabia (1), Syria (157), Thailand (3). *Europe* (22): Russian Federation (3), Ukraine (19). *Americas* (34): Colombia (16), Cuba (8), Ecuador (1), El Salvador (3), Haiti (4), Honduras (1), Puerto Rico (1).

^b^Specific countries of exit: *Africa* (506): Burundi (27), Cape Verde (2), Congo (1), Democratic Republico of Congo (4), Djibouti (1), Egypt (24), Ethiopia (42), Guinea (1), Ivory Coast (13), Kenya (63), Liberia (1), Malawi (13), Mozambique (19), Namibia (12), Niger (1), Rwanda (42), Somalia (1), South Africa (16), Sudan (4), Tanzania (138), Uganda (61), Zambia (18), Zimbabwe (2). *Asia* (178): Afghanistan (11), Indonesia (8), Iran (1), Iraq (23), Jordan (106), Lebanon (5), Malaysia (2), Nepal (7), Pakistan (7), Syria (3), Thailand (5). *Europe* (95): Malta (9), Russian Federation (3), Turkey (64), Ukraine (19). *Americas* (32): Colombia (1), Cuba (2), Ecuador (20), El Salvador (3), Haiti (4), Honduras (1), Puerto Rico (1).

### Screening and Management of Refugees in Their Countries of Exit

Predeparture treatment records were available for 613 (75.5%) refugees. Parasitic testing was not routinely performed in refugees’ countries of exit. On the other hand, presumptive antiparasitic treatment was administered to 600 (97.9%) subjects before departure, as recommended by the CDC [8].

### Characteristics of Refugees With Eosinophilia

One hundred forty-seven (18.1%) of 812 refugees had eosinophilia. The distribution of ages of refugees with or without eosinophilia was similar (*P* = .57) (Table 1). Of individuals with eosinophilia, 46.3% were children (Table 1). The ratio of males to females was significantly greater in individuals with eosinophilia (1.45) than those without eosinophilia (0.93; *P* = .016) (Table 1). Significantly more refugees with eosinophilia originated (85%) or exited (82.3%) from Africa compared with other regions (each *P* < .001) (Table 1).

We defined the severity of eosinophilia as mild (450–1499/μL), moderate (1500–4999/μL), or severe (≥ 5000/μL). The majority (76.9%) of individuals with eosinophilia had mild eosinophilia, followed by moderate (20.4%) and severe (2.7%) (Table 2). While males and females had similar rates of mild and severe eosinophilia, a significantly greater proportion of males had moderate eosinophilia (*P* = .02). There was no significant difference in the severity of eosinophilia when stratified by age (*P* = .41).

**Table 2.** Refugee Demographics Stratified by Severity of Eosinophilia

Characteristic	Mild 450–1499/µL, No. (%)	Moderate 1500–4999/µL, No. (%)	Severe ≥5000/µL, No. (%)	All Refugees, No. (%)
Overall	113 (76.9)	30 (20.4)	4 (2.7)	147 (100.0)
Sex	…	…	…	…
Female	52 (46.0)	6 (20.0)	2 (50.0)	60 (40.8)
Male	61 (54.0)	24 (80.0)	2 (50.0)	87 (59.2)
Age	…	…	…	…
Children (≤18 y)	56 (49.6)	11 (36.7)	2 (50.0)	68 (46.3)
Adult (>18 y)	57 (50.4)	19 (63.3)	2 (50.0)	79 (53.7)

Seventy (47.6%) refugees with eosinophilia were symptomatic at their initial clinic evaluation, although the most frequent symptoms were not necessarily considered to be related to parasitic infections (40.1%) (Table 3). Of the symptoms typically associated with parasitic infections, the most common was abdominal pain (17.1%). None presented with cough, headache, hematochezia, or hematuria. No refugees were diagnosed before departure with parasites that cause eosinophilia.

**Table 3.** Symptoms at Initial Encounter in Symptomatic Refugees With Eosinophilia (n = 70)

Symptom	Positive Frequency, No. (%)
Abdominal pain	12 (17.1)
Pruritus^a^	8 (11.4)
Rash	7 (10.0)
Emesis	5 (7.1)
Diarrhea	4 (5.7)
Nausea	3 (4.3)
Fever	3 (4.3)
Weight loss	2 (2.9)
Fatigue	1 (1.4)
Other symptoms^b^	59 (40.1)

Individuals may have multiple symptoms.

^a^Pruritis with or without dermatosis may indicate onchocerciasis in refugees from endemic regions.

^b^Includes common nonspecific symptoms that are not frequently associated with parasitic infections (eg, back pain, blurry vision, headaches, musculoskeletal pain, tinnitus, vaginal discharge).

Of those with available predeparture treatment information, 112 (97.4%) of 115 refugees with eosinophilia received antiparasitic treatment before departure from their country of exit, which did not differ significantly from those who did not have eosinophilia (488/498, 98%; *P* = .72) (Table 4). There was no difference in severity of eosinophilia between those who did or did not receive prior treatment (*P* = .28). The majority (84.4%) of refugees with eosinophilia were treated ≥15 days before their initial clinic visit in the United States (Table 4). In addition, the majority (92.2%) of pretreated refugees with eosinophilia received 2 or more antiparasitic agents (Table 4). The most common regimens included albendazole, artemisinin-combination therapy, and praziquantel (Table 4). Refugees who were found to have eosinophilia at their initial clinic visit in the United States were more likely to have received artemisinin and praziquantel than albendazole before departure (*P* < .001). The reasons for providers selecting certain antiparasitic agents in the countries of exit were not reported.

**Table 4.** Predeparture Treatment Stratified by Presence of Eosinophilia Among Refugees With Available Treatment Records

	Eosinophilia (n = 115), No. (%)	No Eosinophilia (n = 498), No. (%)
Treatment with antiparasitic agent/s	112 (97.4)	488 (98.0)
Time between predeparture treatment and initial clinic visit	*…*	*…*
0–7 d	1 (0.9)	1 (0.2)
8–14 d	14 (12.2)	47 (9.4)
15–30 d	44 (38.3)	174 (34.9)
31–60 d	51 (44.3)	249 (50.0)
>60 d	2 (1.7)	17 (3.4)
No. of antiparasitic agents	…	…
1	6 (5.2)	69 (13.9)
2	17 (14.8)	179 (35.9)
3	40 (34.8)	104 (20.9)
4	49 (42.6)	136 (27.3)
Pretreatment type	…	…
Albendazole	110 (96.6)	480 (96.4)
Artemisinin combination therapy	97 (84.3)	266 (53.4)
Praziquantel	95 (82.6)	251 (50.4)
Ivermectin	54 (47.0)	278 (55.8)
Amodiaquine	0 (0.0)	7 (1.4)

Individuals may have received >1 treatment type.

### Parasitic Testing of Refugees With Eosinophilia in the United States

All refugees with eosinophilia had at least 1 parasitic test performed at their initial clinic visit in the United States. Stool tests were performed in 99% of individuals as follows: ova and parasite examination (46.6%), multiplex PCR assay (39.0%), or both (13.7%). At least 1 potentially pathogenic intestinal parasite was identified in 43 (29.3%) of 147 individuals. The most prevalent parasites identified were *Giardia duodenalis* (17.0%) and *Blastocystis hominis* (15.0%), although the pathogenicity of the latter is controversial and neither is associated with eosinophilia [9]. Other parasites each accounted for <5% of cases (*Ancylostoma duodenale*, *Entamoeba histolytica*, and *Dientamoeba fragilis*, whose pathogenicity is controversial) (Table 5). Of these parasites, only *Ancylostoma* is known to cause eosinophilia. Five (3.4%) individuals had multiple coinfecting intestinal parasites. There was no significant difference in the frequency of positive stool testing between those who did or did not receive predeparture antiparasitic treatment (*P* = 1.0).

**Table 5.** Parasites Detected in 147 Refugees With Eosinophilia

Diagnostic Test	Positive Frequency, No. (%)
Stool O&P and/or multiplex PCR	…
Protozoa	…
*Giardia duodenalis*	25 (17.0)
*Blastocystis hominis*^a^	21 (14.3)
*Dientamoeba fragilis*^a^	3 (2.0)
*Entamoeba histolytica*	3 (2.0)
Helminths	…
*Ancylostoma duodenale*	1 (0.7)
Nonpathogenic organisms^b^	36 (24.5)
IgG testing	…
*Schistosoma* spp.	23 (15.6)
*Strongyloides stercoralis*	3 (2.0)
Blood smear positive for malaria (*Plasmodium vivax*)	1 (0.7)
Total number of patients positive for any pathogen by stool studies, IgG testing, or blood smear	63 (42.9)
Total number of patients positive for eosinophilia-associated pathogens by stool studies or IgG testing^c^	30 (20.4)

Individuals may be positive for multiple parasites.

Abbreviations: IgG, immunoglobulin G; O&P, ova and parasites; PCR, polymerase chain reaction.

^a^Unclear pathogenic potential.

^b^Presumed nonpathogenic organisms included *Entamoeba coli* (n = 15), *Endolimax nana* (n = 14), *Iodamoeba butschlii* (n = 4), and *Entamoeba hartmanni* (n = 3).

^c^Organisms that have been associated with eosinophilia include *Ancylostoma duodenale, Schistosoma* spp., and *Strongyloides stercoralis.*

Forty-nine (33.3%) refugees with eosinophilia had immunoglobulin G (IgG) serological testing for parasites at the initial visit. Twenty-two (44.9%) tested positive for *Schistosoma* spp. (n = 20) or *Strongyloides stercoralis* (n = 2). Four additional refugees who had serologic testing at a subsequent clinic visit were found to be positive for *Schistosoma* spp. (n = 3) and *Strongyloides* (n = 1). In total, 26 (17.6%) patients tested positive for either *Schistosoma* (15.6%) or *Strongyloides* (2.0%) by serology. Tests for filaria were not performed on any subjects.

Overall, 63 (42.9%) of 147 refugees with eosinophilia tested positive for a parasite by either stool testing, serologic assay, or blood smear, although many of these organisms do not typically lead to eosinophilia.

### Outcomes of Refugees With Eosinophilia

Forty-five (30.6%) refugees with eosinophilia received antiparasitic treatment in the United States (albendazole, ivermectin, and/or praziquantel). Of these, 31 (68.9%) were presumptively treated in their country of departure (2 were not presumptively treated, and 12 had no predeparture information), and 42 (93.3%) had at least 1 parasitic test performed (stool study, serology, and/or blood smear). Attributable causes were *Ancylostoma duodenale* (n = 1), *Schistosoma* spp. (n = 18), and *Strongyloides stercoralis* (n = 3), but positive IgG serology for the latter 2 parasites may have represented remote or adequately treated infection with residual eosinophilia. The majority of refugees with eosinophilia (92.5%) had at least 1 follow-up appointment within 1 year of their initial clinic visit. Sixty-six (44.9%) refugees had no subsequent eosinophil testing. Of the 81 (55.1%) individuals who had repeat blood tests at various time points, eosinophilia resolved in 52 (64.2%) within 1 year. Of these 52 cases, all 36 who had predeparture information available had been presumptively treated. Among the individuals whose eosinophilia resolved, the interval between the first lab test showing eosinophilia and the subsequent test showing a normal eosinophil count ranged from 5 to 365 days, with a median of 103 days (∼3.5 months). Of the 29 (35.8%) refugees whose eosinophilia had not resolved within 1 year, 5 (6.2%) initially had resolution of their eosinophilia but it recurred within 1 year, although the precise causes for the eosinophilia in these and other individuals remained unknown. There were no differences in rates of detection of eosinophilia (*P* = .28) or frequency of follow-up eosinophil testing (*P* = .75) among the 3 refugee clinics.

Three refugees were referred to Infectious Diseases and/or Hematology/Oncology specialists. One of these (0.7%) was diagnosed with *Strongyloides stercoralis*. Five other individuals (3.4%) had alternative diagnoses that could explain their eosinophilia, including eczema, myelofibrosis, and a drug allergy. No patients were diagnosed with *Strongyloides* hyperinfection syndrome or long-term complications, such as infertility or bladder squamous cell carcinoma secondary to *Schistosoma haematobium*, blindness caused by onchocerciasis, or disfigurement due to lymphatic filariasis.

## DISCUSSION

There is a paucity of data on eosinophilia and its utility in diagnosing parasitic infections in resettled refugees in the United States. This retrospective longitudinal analysis of 812 pediatric and adult refugees to the United States is the only comprehensive analysis of this population since 2006 [1]. We found that approximately one-fifth of all subjects evaluated at Rhode Island refugee clinics had eosinophilia on their initial screening test. Ninety-seven percent of refugees with available predeparture treatment information had received at least 1 type of antiparasitic treatment before departure, and the rates and severity of eosinophilia in those who did or did not receive such treatment were similar. More males were found to have eosinophilia (1.5:1 ratio), but there was no association with age, which differs from other publications [1, 7]. Almost half of all refugees with eosinophilia had at least 1 symptom, although symptoms in the majority of cases were not typically associated with parasitic infections. The most common attributable symptom was abdominal pain, which occurred in 17% of individuals with eosinophilia.

All refugees with eosinophilia had at least 1 parasitic test performed at their initial clinic visit at the discretion of the clinical provider. Although there was evidence of a potentially pathogenic parasite in 42.9% of these cases, many of them were protozoa, which are not typically associated with eosinophilia. Some parasites may have been adequately treated predeparture, and the finding of elevated eosinophil counts may have represented residual eosinophilia rather than active infection. The most commonly detected parasites were *Schistosoma* spp., *Giardia duodenalis*, *Blastocystis hominis,* and *Strongyloides stercoralis,* in keeping with prior studies [1–3]. However, *Giardia duodenalis* and *Blastocystis hominis* are not known to cause eosinophilia, and the pathogenicity of *Blastocystis* is controversial. Of the 81 individuals who underwent repeat eosinophil count testing after a median of ∼15 weeks, eosinophilia had resolved in 52 (64.2%). Therefore, eosinophilia in approximately one-third of refugees with subsequent testing persisted despite predeparture presumptive therapy. Although this phenomenon may have represented residual eosinophilia, it could have reflected incomplete or inadequate therapy in at least some of these cases. Notably, in the current series, almost half of refugees with eosinophilia did not have repeat eosinophil testing, which does not align with the CDC recommendation for retesting. Furthermore, one-third had serologic testing for schistosomiasis and strongyloidiasis, which indicates the variability in clinicians’ approaches to patients with eosinophilia. Our findings highlight the importance of educating providers about monitoring eosinophilia until it has fully resolved and pursuing specific diagnostic testing if eosinophilia persists, as recommended by the CDC in Figure 1 on their website [9].

Overall, our findings are in line with those of previous reports [1, 17]. We found a similar percentage of eosinophilia in our refugee population (18.1%) compared with Seybolt et al. (12%), who evaluated refugees at a medical center in Boston from 1998 to 2002 [1], Pavlopoulou et al. (22.7%), who evaluated pediatric immigrants and refugees arriving in Greece from 2010 to 2013 [17], and Janda et al. (18.8%), who evaluated pediatric refugees arriving in Germany from 2016 to 2017 [18]. In contrast, Nutman et al. reported eosinophilia in 50% of newly arrived Indochinese refugees to the Washington, DC, area from 1981 to 1984, which was before the era of predeparture antiparasitic therapy [19].

A study of Barawan Somali refugees conducted in 1997 demonstrated the importance of predeparture treatment of intestinal parasites [12]. In 1999, the CDC recommended that a single dose of albendazole be given to all refugees resettling from Sub-Saharan Africa and Asia; this recommendation was extended to refugees from the Middle East in 2008 [8]. It is likely that this recommendation led to decreases in the prevalence of parasites in resettled refugees since its implementation [20, 21]. *Strongyloides stercoralis* was not as frequently detected in the current study compared with that of Seybolt et al. [1], which may reflect more recent implementation of effective predeparture presumptive therapy in certain locations and the fact that parasitic testing was conducted systematically by Seybolt et al. On the other hand, parasitic testing in the current study was conducted at the providers’ discretion and was not as frequently performed.

We found that predeparture antiparasitic treatment did not have a significant impact on eosinophilia rates at the time of initial testing. This may be explained by the fact that eosinophilia can take weeks to months to resolve despite appropriate treatment, a phenomenon referred to as residual eosinophilia [11]. For that reason, in some cases, elevated eosinophil counts may not reflect active parasitic infections. In the current series, eosinophilia resolved in 64% of the refugees with available repeat blood test results at a median of ∼3.5 months after initial detection. Therefore, our findings support the recommendations of the CDC to recheck eosinophil counts after 3–6 months [9]. If eosinophilia persists, appropriate testing for potential infectious and noninfectious causes is indicated. However, refugees may not be able to access health care easily, which could limit the opportunity to repeat hematological testing. We found that 36% of refugees with eosinophilia did not have repeat testing within 1 year, the time frame of this study, which may reflect the practice of their health care providers or certain circumstances that prevented refugees from returning for further evaluation.

The CDC's testing algorithm for asymptomatic refugees with eosinophilia is an important and useful guideline for health care providers [9]. Additional diagnostic information with links to relevant CDC digital resources for less common parasites that cause persistent eosinophilia may add value to the CDC flowchart. Such parasites include various human and zoonotic nematodes (such as *Toxocara* spp.), filarial spp., tapeworm-related larval cystic diseases (cestodes), and flukes (trematodes). In Box 1, we highlight key clinical pearls for managing refugees with eosinophilia, including how to recognize residual eosinophilia and the importance of developing a standardized approach.

Box 1. **Key Clinical Pearls for Management of Refugees With Eosinophilia**

- Eosinophilia is defined as an absolute eosinophil count >450/µL.

- Residual eosinophilia may persist for 3–6 mo or longer after refugees receive effective predeparture presumptive antiparasitic treatment.

- A standardized CDC-based approach to manage eosinophilia should be established by clinicians who provide health care for refugees as follows:
  - Review information in the Predeparture Electronic Disease Notification System, with particular attention to predeparture presumptive treatment.
  - Refugees who received appropriate predeparture presumptive treatment should have a repeat eosinophil count after 3–6 mo, and diagnostic testing should be pursued if eosinophilia persists.
  - Refugees who did not receive appropriate predeparture presumptive treatment or those with symptoms suggestive of a parasitic disease should undergo diagnostic testing without delay and/or receive targeted empiric therapy with further follow-up.
  - Consider presumptive antiparasitic treatment if the refugees’ access to follow-up health care cannot be assured.
  - Develop systems to contact individuals who need further testing and management, particularly for those whose primary care is elsewhere.
  - Refer to Infectious Diseases if unexplained eosinophilia persists or assistance with diagnostic testing is required. Management is influenced by multiple factors, including country of origin (eg, avoid ivermectin for *Strongyloides* in *Loa loa*–endemic areas).

Abbreviation: CDC, Centers for Disease Control and Prevention.

This study has a number of limitations. Although demographic and predeparture treatment data were collected for all refugees who met eligibility criteria, comprehensive postarrival information was extracted only for individuals with eosinophilia. Therefore, we could not compare rates and results of parasitic tests among those with vs without eosinophilia. As this was a retrospective review, subjects were not investigated or treated for parasites in a systematic manner, and only the most common parasitic causes of eosinophilia were investigated. Positive IgG serologic tests for *Schistosoma* spp. and *Strongyloides stercoralis* do not necessarily prove active infection because antibodies are detectable for prolonged periods regardless of previous treatment.

## CONCLUSIONS

Our findings support the current geographic-specific CDC recommendations to screen for eosinophilia in refugees to the United States regardless of symptomatology [9]. Residual eosinophilia after predeparture presumptive therapy is common and typically resolves within a few months. However, continued monitoring of eosinophil counts and specific diagnostic testing if eosinophilia persists beyond 3–6 months are important to detect ineffectively treated parasites or other treatable conditions [10].

## Supplementary Material

ofae430_Supplementary_Data

## Supplementary Data

Supplementary materials are available at *Open Forum Infectious Diseases* online. Consisting of data provided by the authors to benefit the reader, the posted materials are not copyedited and are the sole responsibility of the authors, so questions or comments should be addressed to the corresponding author.
