# Supplementary material for: A Retrospective Longitudinal Study of Refugees With Eosinophilia at an Academic Center in the United States From 2015 to 2020
Source: Open Forum Infect Dis. 2024 Jul 19;11(8):ofae430. doi: 10.1093/ofid/ofae430 (PMC11327920; doi:10.1093/ofid/ofae430)
Supplement: ofae430_Supplementary_Data [file ofae430_supplementary_data.docx]

**Supplementary Table 1. CDC Recommendations for Overseas Presumptive Parasite Treatment**

| CDC Recommendations for Overseas Presumptive Parasite Treatment | | | | |
| --- | --- | --- | --- | --- |
| **Region** | **Artemether-lumefantrine (malaria)** | **Praziquantel (Schistosoma)** | **Albendazole (soil-transmitted helminths)** | **Ivermectin* (Strongyloides)** |
| **Africa, non-Loaloa areas** | Recommended | Recommended | Recommended | Recommended |
| **Africa, Loa loa areas** | Recommended | Recommended | Recommended | Not Recommended |
| **Asia** | Not Recommended | Not Recommended | Recommended | Recommended |
| **Middle East** | Not Recommended | Not Recommended | Recommended | Recommended |
| **Latin America** | Not Recommended | Not Recommended | Recommended | Recommended |

* If available in country

**(CDC Reference: https://www.cdc.gov/immigrant-refugee-health/hcp/domestic-guidance/intestinal-parasites.html?CDC_AAref_Val=https://www.cdc.gov/immigrantrefugeehealth/guidelines/domestic/intestinal-parasites-domestic.html#cdc_generic_section_7-table-1-recommended-medication-regimen-for-presumptive-treatment-or-treatment-of-identified-parasitic-infections).**
